# Outcome of Arthroscopy in Patients with Advanced Osteoarthritis of the Hip

**DOI:** 10.1371/journal.pone.0113970

**Published:** 2015-01-30

**Authors:** Sachin Daivajna, Ali Bajwa, Richard Villar

**Affiliations:** Spire Cambridge Lea Hospital, Cambridge, CB24 9EL, United Kingdom; Center for Rheumatic Diseases, INDIA

## Abstract

Hip arthroscopy has continued to expand its horizons in treating many conditions other than femoroacetabular impingement (FAI). However, the results of hip arthroscopy are known to be poor if the degree of articular cartilage damage is significant. We wanted to assess, whether the procedure might have a role in the management of young and active patients with advanced osteoarthritis (OA) and whether it should be offered as a treatment modality. 77 consecutive patients with Tönnis grade 2 and 3 osteoarthritis of the hip who had undergone hip arthroscopy were included in the study. Patients' medical notes, plain radiographs and outcome scores (modified Harris hip score (mHHS), non-arthritic hip score (NAHS)) preoperatively and postoperatively at six weeks, six months, one year and annually thereafter, were analysed. 77 patients consisted of 63 men and 14 women with mean follow-up of 2.8 years (2.2 to 4.2) and mean age at surgery of 43 years (19 to 64). The mean preoperative mHHS and NAHS scores were 58 (28 to 87) and 64 (27 to 93) respectively. The mean improvements in both the mHHS and NAHS scores were significant (p = 0.003 and p = 0.0001 for mHHS at one and two years, p = 0.002 and p = 0.0003 for NAHS at one and two years, respectively). There were 34 patients (44%) who required a total hip replacement at mean of 18 months (6 to 48) after hip arthroscopy. We conclude that hip arthroscopy improves outcome scores in 56% of patients with severe OA of the hip (Tönnis grade 2 and 3) for at least two years after surgery. We thus consider the procedure to be a reasonable option for patients with hip OA, although success of the procedure will be less than if undertaken for certain other conditions.

## Introduction

Hip arthroscopy is presently experiencing a large expansion in its use as a conservative surgical procedure. A major indication for its application is in the management of femoroacetabular impingement (FAI), although this has begun to change in recent years as other pathologies have also been recognised as suitable for the operation[1, 2]. However, its role in the management of osteoarthritis (OA) of the hip remains controversial [3–6]. Certainly, arthroscopy for OA in other joints such as the knee has been shown to help patients who have mechanical symptoms [7]. OA of the hip may present clinically with evidence of impingement, pain, and stiffness which might potentially be treated with arthroscopy [8]. Previous reports of its use in patients with OA of the hip have suggested that an improvement in outcome scores can be achieved, although with a 16% to 52% conversion to total hip replacement (THR) [9–12]. However, many patients are reluctant to consider a joint replacement as a suitable option, especially those who are young.

Within our specialist hip arthroscopic practice it is not uncommon for patients, particularly athletes, to ask if hip arthroscopy might be an appropriate choice in the management of their osteoarthritic joint. We thus wanted to look at our results to see, in light of improvements in technique and instrumentation in recent years, whether hip arthroscopy does indeed help patients with OA and, if so, whether it should be offered as a regular treatment modality in such circumstances. We thus present a retrospective analysis of prospectively collected data of patients with Tönnis grade 2 and 3 OA of the hip joint treated by hip arthroscopy and followed for a minimum of two years.

The Tönnis grade is regularly used in our practice in view of its simplicity, no requirement to measure any specific parameter and the ability to use it in the presence of the diverse radiographs encountered when patients may travel many miles, and even cross continents to be seen. The Tönnis grading system is as follows[13]:

0—no signs of osteoarthritis
1—**mild**: increased sclerosis, slight narrowing of the joint space, no or slight loss of head sphericity
2—**moderate**: small cysts, moderate narrowing of the joint space, moderate loss of head sphericity
3—**severe**: large cysts, severe narrowing or obliteration of the joint space, severe deformity of the head

## Methods

The Institutional Review Board at the Spire Cambridge Lea Hospital in Cambridge approved this study and all patient records were anonymised and de-identified prior to analysis. We identified 77 consecutive patients with Tönnis grade 2 and 3 OA of the hip who had undergone hip arthroscopy and included them in this study. Osteoarthritis was diagnosed when features of loss in joint space, osteophytes, sclerosis or subchondral cysts were seen on a plain radiographs. Patients had symptoms of hip pain, which did not respond to conservative modes of management. Patients’ medical notes, plain radiographs and outcome scores (modified Harris hip score (mHHS), non-arthritic hip score (NAHS)) preoperatively and postoperatively at six weeks, six months, one year and annually thereafter, were analysed. The scores were obtained by using a postal questionnaire.

The modified Harris hip score is a modification of the Harris hip score, which was originally developed for use in total hip arthroplasty patients[14]. The modified version includes a clinical assessment of pain (44 points) and function (47 points) for a total score out of 91 points, with a higher score indicating greater function and less pain. The Non-Arthritic Hip Score (NAHS) is a validated disease-specific questionnaire, consisting of 20 questions, divided into four domains: pain, symptoms, physical function and participation[15]. Items are scored from 0–4, and added together for an overall total score. Three items on the instrument assess sports-related activities. A higher score represents a higher level of physical function and less pain and symptoms.

The Tönnis grading on plain radiographs was used to identify this cohort of patients as this is widely used in practice and is perhaps more practical in the outpatient, clinical situation than any other system that may require the physical measurement of radiographs or other imaging techniques. The senior author (RNV) operated on all cases and employed the lateral position using a Smith and Nephew (S&N, Memphis, TN) Hip Positioning System[16]. The causes of osteoarthritis and the main procedures performed during arthroscopy are presented in Tables 1 and 2, respectively. There was no regenerative cell therapy performed. All patients had local anaesthetic infiltration into the portals at the end of surgery and 60mg/3ml of hyaluronic acid (Durolane; Smith & Nephew, York) instilled into the joint. Data collected for this study were entered into a Statistical Package for the Social Sciences (SPSS; IBM, New York, NY) statistical program. We used Student’s t-test to analyse the change in the mHHS and NAHS scores between that found pre-operatively and the scores at six weeks, six months, one year and thereafter every year after surgery. We used univariate analysis (Fisher’s exact test) to analyse the effect of age on the necessity for a subsequent total hip replacement. We considered a p value < 0.05 as significant.

**Table 1:** Etiology of osteoarthritis in patients.

**Causes of osteoarthritis (OA)**	**Number**
Primary OA	38
Cam/ pincer femoroacetabular impingement	34
Dysplasia	2
Avascular necrosis	1
Osteochondromatosis	1
Slipped upper femoral epiphysis	1

**Table 2:** Procedures carried out during hip arthroscopy.

**Procedure**	**Number**
Partial acetabular labrectomy + chondroplasty + excision of osteophytes /cam lesion	58
Rim excision + partial acetabular labrectomy + chondroplasty + excision of osteophytes/ cam lesion	14
Removal of loose bodies in addition to chondroplasty and excision of osteophytes	4
Rim excision + labral graft + chondroplasty + cyst excision	1

## Results

Of the 77 patients there were 63 men and 14 women. The mean follow-up was 2.8 years (2.2 to 4.2) and the mean age at surgery was 43 years (19 to 64). The mean preoperative mHHS and NAHS scores were 58 (28 to 87) and 64 (27 to 93), respectively. This improved to 66 (28 to 91) and 74 (30 to 100), respectively, at the one-year follow-up and to 72 (47 to 91) and 77 (59 to 95) by two years. The mean improvements in both the mHHS and NAHS scores were significant (p = 0.003 and p = 0.0001 for mHHS at one and two years, p = 0.002 and p = 0.0003 for NAHS at one and two years, respectively). There were 34 patients (44%) who required a total hip replacement at mean of 18 months (6 to 48) after hip arthroscopy. The duration between hip arthroscopy and hip replacement in patients is shown in Table 3.

**Table 3:** The duration between hip arthroscopy and total hip replacement.

**Time from hip arthroscopy to THR**	**Number**
0–6 months	3
6–12 months	6
12–24 months	10
24–48 months	14
> 48 months	1

We also compared the incidence of THR in patients aged < 50 years (n = 60) compared with those aged ≥ 50 years (n = 17) but found no significant difference between the two groups (p = 0.79).

## Discussion

Hip arthroscopy has improved outcomes and relieved the symptoms of FAI across multiple studies and there are now good outcome and follow-up data [3, 17, 18]. This has led many patients with OA of the hip to present to our specialist practice seeking a possible arthroscopic solution to their problem. Such patients are frequently athletically inclined and wish to conserve their hip joint for as long as possible, even if they are made aware that conversion to an arthroplasty may one day be required [19, 20]. However, multiple other studies have suggested that outcomes are much poorer in the presence of chondral damage and/or osteoarthritis and have led to the belief that OA is not a good indication for hip arthroscopic surgery[21–23]. Experience with other joints has also supported this stance[24, 25]. Nevertheless, in response to the modern-day improvements in both technique and instrumentation related to hip arthroscopy, within our own practice it seemed sensible to reappraise this view. We also consider that within a busy outpatient clinic, there is little time, or even inclination to precisely measure all imaging in order to establish whether or not surgery might be beneficial. Surgical decision-making is a combination of a clinician’s viewpoint and experience balanced against a patient’s desires and needs. Our finding that 34 patients (44%) required a total hip replacement at mean of 18 months (6 to 48) after surgery is better than some earlier studies but worse than certain others[11, 26]. However, 44% failure implies 56% success. Furthermore, we had no complications in these patients and the results appeared not to be influenced by age, a feature reported elsewhere by others [22, 27].

There are a few limitations to our study. We instilled Durolane (hyaluronic acid) intraarticularly at the end of each procedure. This may affect our results compared to previous studies that may not have used this material. The reason for use of this product was mainly to reduce pain in the immediate postoperative period. Also, the procedures carried out for each patient may differ due to varied pathology. Consequently, we could not standardise the procedure applied to each patient. However, all techniques used were aimed to repair labral and articular cartilage damage and improve the motion of the joint by reducing the impingement. Our results are at mean of 2.8 years. Further follow-up would be required to determine the survival of this procedure.

In light of these findings, our own policy in respect of the arthroscopic management of the osteoarthritic hip has changed. Whereas once we saw it as a contraindication, we now present the chances of success to the patient. Is a 56% chance of symptomatic improvement for a mean of two years acceptable in the presence of established osteoarthritic change? Some patients will regard this as acceptable success while others will not. Ultimately it is the patient who must decide.
